# The stability region of the *Streptomyces lividans* plasmid pIJ101 encodes a DNA-binding protein recognizing a highly conserved short palindromic sequence motif

**DOI:** 10.3389/fmicb.2014.00499

**Published:** 2014-09-22

**Authors:** Lina Thoma, Edgardo Sepulveda, Annette Latus, Günther Muth

**Affiliations:** Mikrobiologie/Biotechnologie, Interfakultäres Institut für Mikrobiologie und Infektionsmedizin Tübingen IMIT, Eberhard Karls Universität TübingenTübingen, Germany

**Keywords:** rolling-circle replication, sso, TraB, FtsK, plasmid transfer

## Abstract

Conjugation is a driving force in the evolution and shaping of bacterial genomes. In antibiotic producing streptomycetes even small plasmids replicating via the rolling-circle mechanism are conjugative. Although they encode only genes involved in replication and transfer, the molecular function of most plasmid encoded proteins is unknown. In this work we show that the conjugative plasmid pIJ101 encodes an overlooked protein, SpdA2. We show that SpdA2 is a DNA binding protein which specifically recognizes a palindromic DNA sequence (*sps*). *sps* is localized within the *spdA2* coding region and highly conserved in many *Streptomyces* plasmids. Elimination of the palindrome or deletion of *spdA2* in plasmid pIJ303 did not interfere with conjugative plasmid transfer or pock formation, but affected segregational stability.

## Introduction

Streptomycetes, mycelial growing gram-positive soil bacteria with a complex life cycle are the most important producers of antibiotics and other secondary metabolites (Aigle et al., 2014). Since the antibiotic biosynthetic gene clusters not only encode the biosynthetic enzymes for the synthesis but also resistance mechanisms to protect the producer from its own antibiotic, streptomycetes are regarded as the original source of antibiotic resistance genes (Thaker et al., 2013). By horizontal gene transfer (HGT) the resistance genes probably found their way into pathogenic bacteria, causing major health problems.

Many mobile genetic elements have been described in the genus *Streptomyces* (Vogelmann et al., 2011a). These include small plasmids replicating via the rolling-circle mechanism (Kieser et al., 1982; Kataoka et al., 1994; Muth et al., 1995; Servín-González et al., 1995; Reuther et al., 2006), larger low copy number plasmids (Schrempf and Goebel, 1977; Wohlleben and Pühler, 1987; Haug et al., 2003), plasmid-phages that can propagate and conjugate as episomal DNA but can also produce infective phage particles (Chen et al., 2012), and linear plasmids of several hundred kbp in size (Kinashi et al., 1987). Moreover, *Streptomyces* chromosomes often contain several copies of integrated chromosomal elements that can be excised and conjugally transferred to other bacteria where they integrate again, usually within a specific tRNA gene (Boccard et al., 1988; te Poele et al., 2008).

In contrast to the majority of plasmids from other bacteria, *Streptomyces* plasmids, with rare exceptions, do not carry antibiotic resistance genes or other traits that might be advantageous under certain environmental conditions (Sepulveda et al., 2011). This is in particular apparent in the small multi-copy plasmids which encode around 10 proteins, involved only in replication and conjugative transfer (Vogelmann et al., 2011a). The replication initiator protein Rep initiates rolling-circle replication at the double-stranded origin *dso* (Espinosa et al., 1995), while the conversion of the circular single-stranded replication intermediate into a double-stranded plasmid molecule at the minus-origin *sso* is directed by host factors (Kramer et al., 1997). Inactivation of the *sso* results in the accumulation of large amounts of single-stranded DNA, a reduced copy number and increased segregational instability (Deng et al., 1988).

*Streptomyces* conjugation is distinguished from the classical conjugation via a type IV secretion system and requires a single plasmid encoded protein, the FtsK homolog TraB (Pettis and Cohen, 1994; Thoma and Muth, 2012). During conjugation, hexameric TraB assembles at the plasmid localized *clt* locus, recognizing 8 bp TRS repeats and directs the transfer of a double-stranded plasmid molecule. Chromosomal markers are probably mobilized by the interaction of TraB with chromosomal *clt*-like sequences present on *Streptomyces* chromosomes (Vogelmann et al., 2011b).

In the recipient, the newly transferred plasmid is thought to spread across the septal cross wall resulting in the rapid colonization of the recipient colony by the incoming plasmid (Grohmann et al., 2003). Under certain mating conditions, when few donors are plated with an excess of recipients, plasmid spreading is manifested by the formation of inhibition zones with a diameter of up to 3 mm, called pocks. These pock structures represent temporary growth retardation zones and indicate areas in the mycelial lawn where a recipient has obtained a plasmid by conjugation (Hopwood and Kieser, 1993). Pock formation depends on 3–6 *spd* genes. None of the predicted Spd proteins shows any similarity to a functionally characterized protein. The *spd* genes of different *Streptomyces* plasmids are highly diverse in size and many plasmids contain unique *spd* genes that do not show any sequence similarity to *spd* genes from other plasmids (Vogelmann et al., 2011a). The integral membrane protein SpdB2 of the *S. venezuelae* plasmid pSVH1 was shown to have a non-specific DNA binding activity and to oligomerize to larger structures (Tiffert et al., 2007). Interestingly, it has also been demonstrated that SpdB2 interacts with TraB and Spd79, suggesting the existence of a multi-protein machinery involved in plasmid spreading. No further information on other *spd* genes is available although it is known that the inactivation of a single *spd* gene reduces pock size (Kieser et al., 1982; Kataoka et al., 1994; Servín-González et al., 1995).

One of the best studied *Streptomyces* plasmids is pIJ101 from *S. lividans* (Kieser et al., 1982). pIJ101 has a molecular size of 8830 bp and was the first conjugative plasmid from a Gram-positive bacterium to be sequenced in its entity (Kendall and Cohen, 1988). A replication initiator gene, a single-stranded origin *sso* (*sti*), an essential transfer gene *traB* (*kilA*) and several spread genes have been identified by subcloning experiments. Expression of the transfer and spreading genes is controlled by two transcriptional repressors, KorA and KorB (Stein and Cohen, 1990).

Here we report the characterization of a small ORF (*spdA2*) that was missed in the original sequence annotation. A homolog of this ORF was previously shown to be involved in intramycelial plasmid spreading of the *S. nigrifaciens* plasmid pSN22 (Kataoka et al., 1991, 1994). Our data however suggest that SpdA2 of pIJ101 is not involved in intramycelial plasmid spreading but affects plasmid stability by binding to a palindromic sequence within the *spdA2* gene.

## Materials and methods

### Computer assisted analysis

To identify homologs of SpdA2 proteins, we searched the non-redundant database at NCBI using PSI-BLAST (Altschul et al., 1997). Protein domain search was performed with Pfam v23.0 (Finn et al., 2010).

### Bacterial strains, growth conditions, and DNA manipulation

Cultivation of strains and procedures for DNA manipulation were performed as previously described for *E. coli* (Sambrook and Russel, 2001) and *S. coelicolor* (Kieser et al., 2000). For plasmid manipulation and protein overproduction we used *Escherichia coli* strains XL1blue (Bullock et al., 1987) and BL21(DE3) pLysS (Merck Millipore), respectively. For mating experiments we used *S. lividans* strains TK64 and TK54 (Hopwood et al., 1983) transformed with the required plasmids. Antibiotics were used at the following concentrations: ampicillin 150 μg/ml; kanamycin 50 μg/ml; thiostrepton 25 μg/ml; apramycin 50 μg/ml. Plasmids are listed in Table 1 and primers in Table 2.

**Table 1:** **Strains and plasmids used in this work**.

**Plamid**	**Characteristics**	**References**
*S. lividans* TK54	*his-2, leu-2, spc-1*	Hopwood et al., 1983
*S. lividans* TK64	*pro-2, str-6*,	Hopwood et al., 1983
*S. lividans* TK64::pSET152	*pro-2, str-6, aac(IV)3*	This work
*E. coli* XL1blue	*endA1, gyrA96*(nal^R^), *thi-1, recA1, relA1, lac, glnV44*, F′[::Tn*10 proAB*^+^, *lacI^q^*, Δ (*lacZ*)M15], *hsdR17*(r^−^_K_ m^+^_K_)	Bullock et al., 1987
BL21(DE3) pLysS	F^−^, *ompT, gal, dcm, lon, hsdS_B_*(r^−^_B_ m^−^_B_) λ (DE3), pLysS(cm^R^)	Merck, USA
pEB211	Fusion of pSVH1 with pK18 via NheI sites	Reuther et al., 2006
pIJ101	Conjugative *Streptomyces* plasmid	Kieser et al., 1982
pIJ303	pIJ101 derivative with a thiostrepton resistance gene	Kieser et al., 1982
pIJ303ΔspdA2	pIJ303 derivative with a *spdA2* deletion	This work
pIJ303spdA2c	pIJ303Δ spdA2 complemented with *spdA2* in NdeI/HindIII sites	This work
pIJ303-sps	pIJ303Δ spdA2 complemented with *spdA2*-*sps* in NdeI/HindIII sites	This work
pJOE2775	*E. coli* expression vector, *bla*, P_rham_ promoter, C-terminal His-tag	Altenbuchner, personal communication
pSET152	Integrative pMB1derivative, PhiC31 *int, attP, aac(IV)3*,	Bierman et al., 1992
pSSH01	pJOE2775 with *spdA2* cloned in NdeI/BamHI	This work
pSSH02	pMK-RQ with *spdA2*-*sps*	This work

**Table 2:** **Oligonucleotid primer used in this work**.

**Name**	Sequence^*^
SpdA2-101Nde	AA**CATATG**AGCACCACCACC
SpdA2-101Bam	AA**GGATCC**AGGGGTTTGCGGGTC
UpspdA101	CACTTCGCACTAACTC
Sps1-101rev	GAAGGCTGCTGCATTT
SpdA+1	GG**CATATG**ACGGCTAGGGTCG
SpdAloB	AA**AGATCT**CGCGTCCGGCTGCCCCTG
SpdA-400	AA**GAATTCAATATT**GAAGACAGGAGAGGA
TraRNde	AA**CATATG**ACTTCGACACAGAGC
TraRhis	AA**AAGCTT**TCAGTGGTGGTGGTGGTGGTGGACCTCC
	AGCTCGTA
303DelAu	AGGA**TCTAGA**GGCGCCCGCCCTCGAA
303DelAl	TGCT**CATATG**AATTCACCGTACGCGGCACG
303DelBu	GCCC**GGTACCAAGCTT**ACCCGCAAACCCCTT
303DelBl	TGGC**CTCGAG**AGGGACGCGGGCGA
tsr-146fw	AAGATCGTCGGGAACATCGG
tsr-146rev	ACGGGAAGGGAGAAGACGTA
Slxre-146up	TCCAGGCGAGTCTTCCAGTA
Slxre-146lo	ACACGGTGATGTACGAGCAT

* *Bold letters indicate restriction sites*.

### Over-expression and purification of SpdA2

To generate a C-terminally His-tagged SpdA2 derivative, the *spdA2* coding sequence was amplified from pIJ101 plasmid DNA using primers SpdA2-101Nde and SpdA2-101Bam, which contain custom-made NdeI and BamHI sites, respectively. After digestion with the appropriate enzymes, the PCR product was ligated into pJOE2775 giving rise to plasmid pSSH01 which was sequenced and then introduced into *E. coli* BL21. Cultures were grown in 500 ml of Luria-Bertani medium at 29°C to an *A*_600_ of 0.4. At this point, 20% rhamnose was added to a final concentration of 0.2%; cells were harvested 4 h later and the cell pellet was resuspended in 5 ml of ice-cold extraction buffer (100 mM Tris HCl, 0.3 M NaCl, pH 7.6). Cells were broken by three cycles of thawing and freezing, followed by three passages through a French press (Thermo Spectronic Instruments). The extract was centrifuged at 10°C for 10 min at 7800 × *g* to obtain the cell-free fraction. To purify His-tagged SpdA2, a 1-ml Ni^2+^ affinity column (Ni-NTA Super flow, IBA) was equilibrated with extraction buffer containing 10 mM imidazole. Ten milliliters of cell extract was passed and then the column was washed with 10 volumes of the same buffer. His-tagged SpdA2 was eluted in 3 steps using extraction buffer containing 50, 100, and 150 mM imidazole.

### *In-vitro* synthesis of DNA

*In-vitro* synthesis of the FA2-sps fragment was performed by Mr. Gene GmbH (actually GeneArt, Invitrogen). Sequence was designed to be identical to *spdA2* with a C-terminal His-tag encoding sequence but lacking the two *sps* palindromes. The synthesized gene was delivered in plasmid pSSH02, (a proprietary pBluescript+SK derivative).

### Emsa analyses

DNA regions were amplified by PCR using the following primer pairs (Table 2): from pIJ101 DNA, SpdA2-101Nde/SpdA2-101Bam (fragment FA2) and UpspdA2-101/Sps1-101rev (fragment F1sps); from pEB211 DNA, SpdA+1/SpdAloB (fragment FSpdA), SpdA-400/SpdAloB (fragment FSpdA_up_) and TraRNde/TraRhis (FTraR_pSVH1_); from pSSH02 DNA, SpdA2-101Nde/SpdA2-101Bam (Fragment FA2-sps). Products were electrophoresed on a 1.5% agarose gel and purified by gel extraction using the Illustra™ GFX™ PCR DNA and Gel Band Purification Kit (GE Healthcare). Purified SpdA2-His protein was incubated with the desired DNA fragments for 30 min at room temperature in binding buffer (20 mM NaH_2_PO_4_, 300 mM NaCl, 50 mM KCl, 0.3 mM MgCl_2_, 0.5 mg/ml BSA, pH 8). As a control for specificity, the FTraR_pSVH1_ fragment was included in the binding reaction mixture. Binding reaction mixtures were electrophoresed on a 6% Tris-acetate-polyacrylamide gel at 60 V for 1.5 h and stained with ethidium bromide for visualization with UV light.

### Construction of plasmids pIJ303ΔspdA2, pIJ303-sps, and pIJ303spdA2c

To construct pIJ303Δ spdA2 we performed an XL-PCR (KAPA Long Range DNA Polymerase, Kapa Biosystems) with primers 303DelAl/303DelBu (Table 2). The PCR product was purified, ligated and introduced into *S. lividans* TK54 by protoplast transformation. Transformants were selected with thiostrepton and screened by colony PCR using oligonucleotides 303DelAu/303DelBl searching for a product of 1860 bp as opposed to the 2229 bp fragment produced by the wild type pIJ303 plasmid. The PCR product of the expected size was sequenced for further confirmation of the *spdA2* deletion. To construct plasmids pIJ303spdA2c and pIJ303-sps, we digested plasmids pSSH01 and pSSH02 with NdeI/HindIII and purified the FA2 and FA2-spsfragments, respectively. Next, these fragments were separately ligated into NdeI/HindIII digested pIJ303Δ spdA2 and transferred to *S. lividans* TK54. Transformants were screened by colony-PCR using oligonucleotide primers 303DelAu/303DelBl. Further validation was provided by a digestion of the PCR products with BamHI which resulted in fragments of 949, 864 and 416 bp due to an extra BamHI site located within the cloned *spdA2* allele as opposed to a wild type product that results in a 1813 bp and a 416 bp fragment.

### Plasmid stability assays

Following transformation of *S. lividans* TK54 the presence of plasmids was determined by alkaline lysis. A single colony was homogenized and plated onto MS agar and incubated at 30°C for 5 days. Four replicas were made. After one, two and three rounds of sporulation, spores were harvested and plated to single colonies on LB agar. Then, 100 colonies were picked in parallel on LB-thiostrepton and on antibiotic free medium to calculate the frequency of plasmid loss. For statistical tests using IBM SPSS Statistics, version 22 (IBM Corp. in Armonk, USA), arcsine transformation was performed on the obtained frequencies, and the transformed frequencies for each sporulation cycle were compared using One-Way anova. The level of significance was adjusted with Bonferroni correction for multiple comparisons.

### Determination of relative plasmid copy numbers

To examine differences in the copy number of the pIJ303 derivatives, qPCR was performed. Total DNA was extracted from equal amounts of mycelium using the peqGOLD Bacterial DNA Kit (Peqlab, Germany) according to the manufactures instructions. For each strain total DNA was extracted from five independent cultures which were grown over night under antibiotic selection. DNA concentration was measured with an UV-Vis Spectrophotometer NanoDrop ND-1000 (Peqlab, Germany) and 0.6 ng DNA were used for each qPCR reaction. To detect plasmid DNA, we used primers tsr-146fw and tsr-146rev that amplified a 146 bp fragment of the thiostrepton resistance gene *tsr*. As a reference, a 146 bp fragment of the *S. lividans* chromosomal *xre* gene was amplified with primers Slxrs-146up and Slxre-146lo. Samples for qPCR were prepared using Maxima SYBR Green qPCR Master Mix (2×) (Thermo Fisher Scientific, USA) in a total volume of 20 μl using 0.3 M of each primer. Amplification and monitoring was performed using an IQ5 iCycler (Biorad, USA). PCR cycling conditions were the following: 10 min at 95°C, 40 cycles of 95°C for 15 s, 56.8°C for 30 s, 72°C for 30 s for all reactions. Subsequently a melt curve analysis was performed to confirm the specificity of the PCR products. Cycle threshold (Ct) values were determined automatically by the IQ5 software after automated base line subtraction. Each biological replicate was analyzed in two independent qPCR experiments in triplicates. PCR efficiencies were tested for both primer pairs by the dilution method (Higuchi et al., 1993; Rasmussen, 2001) using total DNA extracted from TK54(pIJ303) as a template and gave values of 93.1% for *tsr* amplification and 94.9% for *xre* amplification, respectively. Since the PCR efficiency to detect plasmid DNA and chromosome are not the same no absolute determination of the plasmid copy number was possible.

PCR efficiencies and Ct values were used to calculate the relative quantity of the different plasmid derivates by the Pfaffl mathematical model (Pfaffl, 2001). Values were calculated according to the following formula:

$$relative\;plasmid\;quantity=\frac{E_{tsr}^{\left(Ct(pIJ303,tsr)-Ct(test,tsr)\right)}}{E_{xre}^{\left(Ct(pIJ303,xre)-Ct(test,xre)\right)}}$$

Statistical significance was analyzed with IBM SPSS Statistics, version 22 (IBM Corp. in Armonk, USA) applying the Mann–Whitney *U*-test with a significance level of 0.05.

### Analyses of transfer frequencies and pock formation

10^7^ spores of plasmid carrying *S. lividans* TK54 (resistant to spectinomycin and thiostrepton) were mixed with 10^7^ TK64::pSET152 (resistant to streptomycin and apramycin) spores and plated on R5 plates, supplemented with 20 μM CuCl_2_ to stimulate spore formation. After 5 days of growth at 30°C, spores were harvested and titered on LB-apramycin (recipient titer) and on LB-thiostrepton/apramycin (transconjugant titer). Transfer frequency is the transconjugant titer, divided by the recipient titer in percentage. For statistical tests using IBM SPSS Statistics, version 22 (IBM Corp. in Armonk, NY, USA), a Mann–Whitney *U*-test was applied with a significance level of 0.05.

Pock formation was analyzed by plating ~10^6^ spores of plasmid free TK64::pSET152 (resistant to streptomycin and apramycin) on R5 plates containing 20 μM CuCl_2_. Subsequently dilutions of *S. lividans* TK54 containing the different pIJ303 derivatives (resistant to spectinomycin and thiostrepton) were streaked across the lawn. After 2 days of incubation at 30°C the plates were inspected for pock structures. After 7 days the fully sporulated plates were replica plated onto LB-agar containing thiostrepton and apramycin to allow only transconjugant growth within the pock area.

## Results

### pIJ101 encodes a SpdA homolog

**Figure 1:**
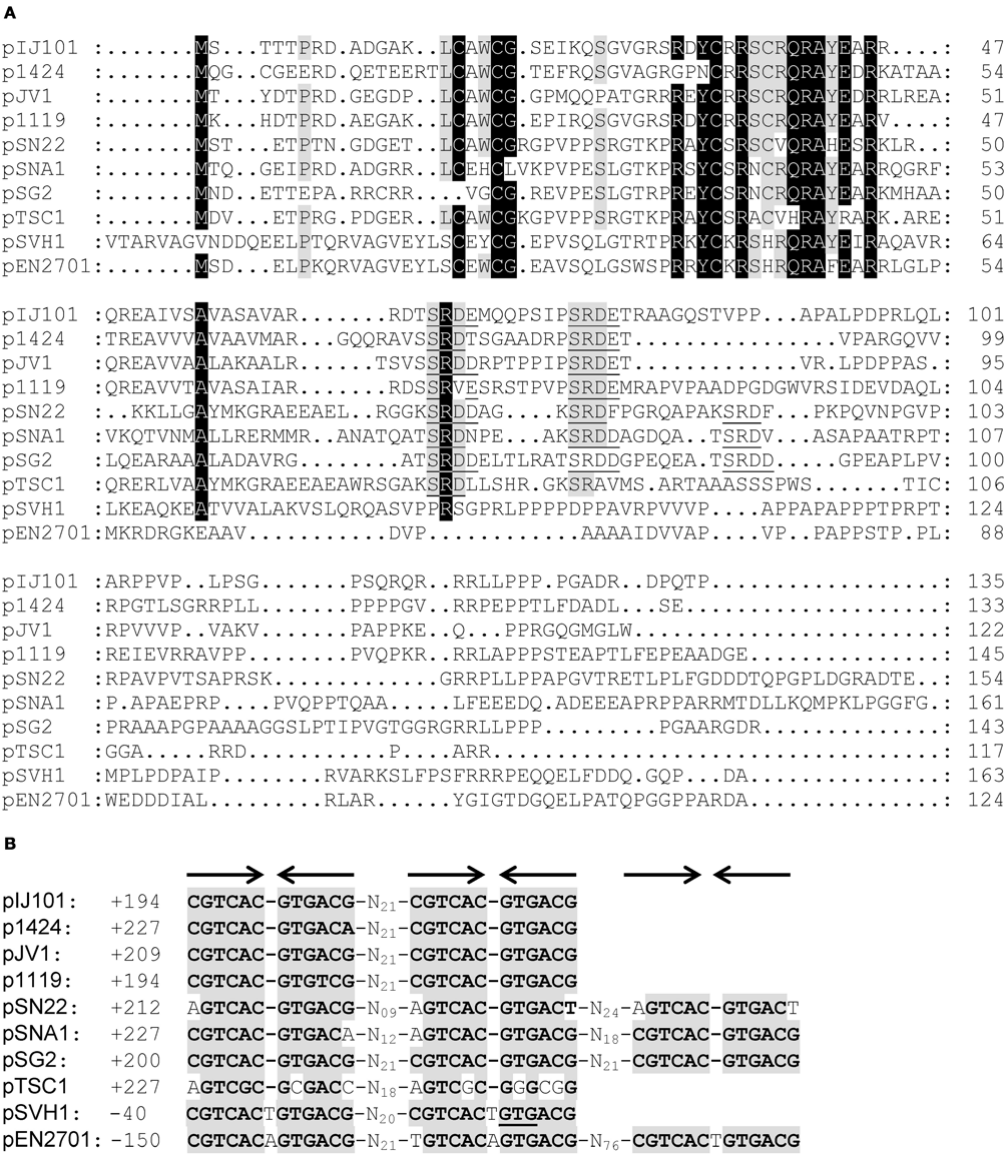
**Association of *spdA* homologs with a conserved palindromic DNA sequence motif on *Streptomyces* plasmids**. Alignment (CLUSTALW) of predicted SpdA proteins encoded by different *Streptomyces* plasmids **(A)**. Conserved residues are indicated by shading (black >90%, gray >70% identity). Gaps were manually introduced to improve the alignment. The SRDD/E aa motif corresponding to *sps* is underlined. Multiple-sequence alignment of ***s****pdA* **p**alindromic (arrows) DNA **s**equence (*sps*) motifs associated with *spdA* genes **(B)**. Conserved residues are indicated by gray boxes. ± Indicates position from the first base of the start codon. On pSVH1, the second palindrome overlaps with the translational start codon (underlined) of *spdA*.

As previously suggested (Servín-González et al., 1995), the non-coding region between *rep* and the single-stranded origin *sso* of plasmid pIJ101 contains an ORF. Its predicted 135 amino acid product with a molecular weight of approximately 14.7 kD shows significant sequence similarity to SpdA proteins which are encoded by various *Streptomyces* plasmids (Figure 1A). Although overall similarity is very low, all SpdA homologs have a conserved sequence at their N-termini with an identity of up to 58% over a region of 38 amino acids.

Strikingly, the *spdA* genes also contain a highly conserved 12 bp palindromic nucleotide sequence which is repeated 2–3 times, (Figure 1B). This sequence motif which was firstly described for *spdA* of pJV1 (Servín-González et al., 1995) corresponded with a SRDD/E-X_3−8_-SRDD/E amino acid motif within most SpdA proteins with the notable exceptions of plasmids pSVH1 (Reuther et al., 2006) and pEN2701 (Coombs et al., 2003). pEN2701 contains three palindromes upstream of *spdA*, whereas in plasmid pSVH1 the second palindrome overlaps with the translational start codon of *spdA* (Figure 1B). Interestingly, the palindromes of plasmid pSVH1 and pEN2701 slightly differ in their sequence from the palindromes of the other plasmids and contain a T (A) insertion at the center of the palindrome (Figure 1B).

Since *Streptomyces* plasmids and in particular *spd* genes are highly variable, the detection of a conserved sequence motif on *Streptomyces* plasmids was unexpected. This palindromic repeat was named *sps* for ***s****pdA*-**p**alindromic-**s**equence. Because a gene located near *spdB* of pIJ101 has already been named *spdA* (Kendall and Cohen, 1988), we decided to name the *spdA*-like ORF as *spdA2* to avoid confusion.

### *spdA2* encodes a DNA-binding protein

When we screened the predicted SpdA2 amino acid sequence for the presence of specific protein domains to elucidate its molecular function, we found hits for a zinc finger motif. Moreover, a BLAST search also retrieved several putative regulatory proteins from actinomycetes in which the region of similarity was located within the potential DNA-binding domain corresponding to the conserved region at the N-terminus. These results suggested that SpdA2 may possess a DNA-binding activity. Palindromic sequences are common binding sites for DNA-interacting proteins (Ohno, 1990) making the palindromes found in the *spdA2* region a strong candidate for a potential target sequence of the protein.

**Figure 2:**
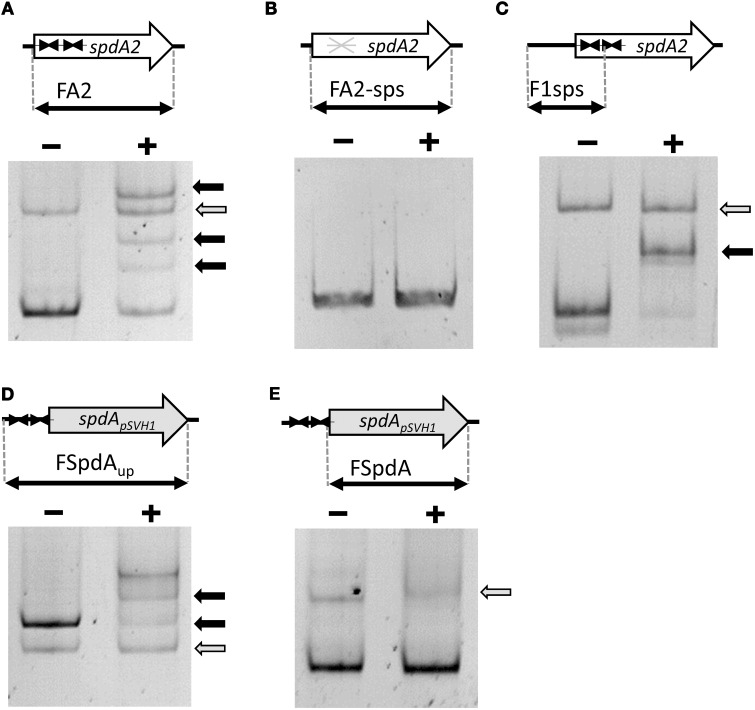
**Electrophoretic mobility shift assays demonstrating binding of SpdA2-His to the palindromic sequence motif *sps***. Different PCR fragments, as schematically illustrated, were incubated with ~2 pmol purified SpdA2-His protein (+) or without SpdA2-His (−), run on a 6% Tris-acetate polyacrylamide gel and stained with EtBr. The gray arrow indicates the band corresponding to a PCR fragment (FTraR_pSVH1_) added as a negative control for binding specificity. Black arrows mark retarded DNA fragments. Interaction of SpdA2-His with the FA2 fragment resulted in several retarded bands **(A)**. Whereas SpdA2-His did not interact with the FA2-sps fragment **(B)**, SpdA2-His shifted fragment F1sps, containing a single palindromic *sps* sequence **(C)**. SpdA2-His also bound to the PCR fragment FSpdA_up_, containing a T insertion within the palindromic sequence *sps* **(D)**, but did not recognize FSpdA, lacking *sps* **(E)**.

To test these hypotheses we over-expressed and purified SpdA2 fused to a C-terminal His-tag and performed electrophoretic mobility shift assays (EMSA) against a PCR product (FA2) of the coding region of *spdA2* of plasmid pIJ101. When SpdA2 was added, even at very low concentration, we were able to detect retarded bands that clearly demonstrated that SpdA2 is a DNA-binding protein (Figure 2A, black arrows).

### SpdA2 binds specifically to the *spdA2* palindromic repeat *sps*

To study the proposed role of the palindromic sequence in SpdA2 binding in more detail, we performed additional EMSAs with different PCR fragments (Figure 2). In all experiments a non-specific fragment (FTraR_pSVH1_; Figures 2A–E, gray arrows) was included as a negative control to discriminate specific DNA-binding activity of SpdA2-His from non-specific DNA interaction.

**Figure 3:**
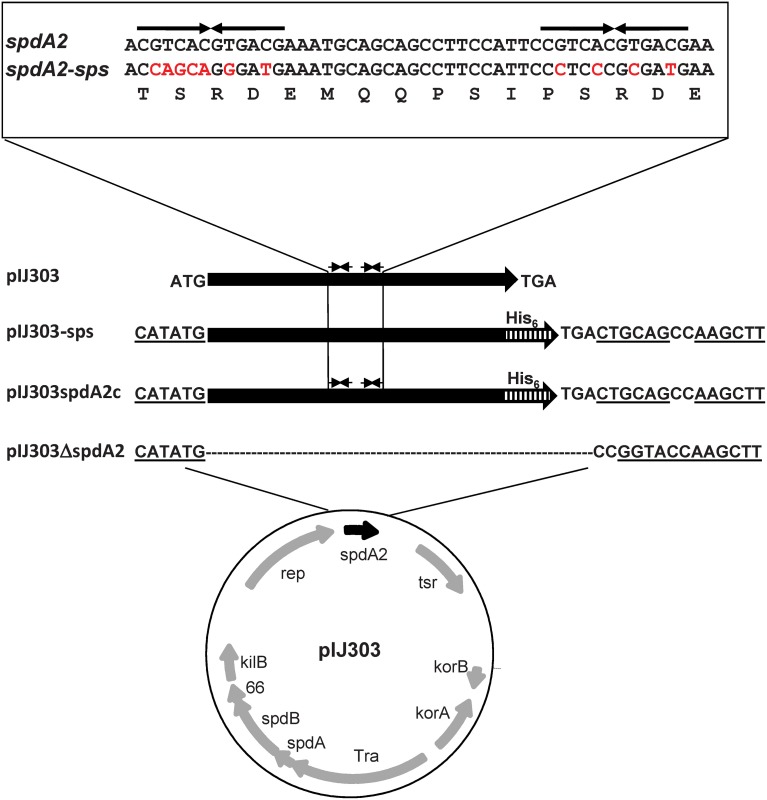
**Schematic maps of pIJ303 derivatives used to elucidate the function of *spdA2***. The *spdA* homolog *spdA2* (black arrow) of plasmid pIJ303 lies downstream of *rep*. In pIJ303ΔspdA2, *spdA2* was replaced (see Materials and Methods) by a linker sequence containing restriction sites (underlined). The NdeI site is coincident with the original *spdA2* start codon. The complementing plasmid pIJ303spdA2c contains *spdA2* with a C-terminal His-tag encoding sequence (hatched bar), while pIJ303-sps contains a synthetic *spdA2-His* gene, where the palindromic sequences (upper part, thin arrows) were removed by base substitutions (indicated by red color) that did not alter the amino acid sequence of SpdA2 (lower line).

Whereas SpdA2-His binding to fragment FA2, which contains both *sps* sites, resulted in several shifted bands (Figure 2A, black arrows), the mutated FA2-sps fragment, where the palindromic sequence was changed by base substitutions (Figure 3), showed no retardation (Figure 2B). Therefore, it is most likely that the palindromes represent the binding site of SpdA2.

In order to analyse whether both palindromes are required for SpdA2 binding, a fragment containing a single *sps* site (F1sps) was amplified from plasmid pIJ101 and tested for SpdA2-His binding. A single retarded band was observed upon addition of SpdA2-His (Figure 2C, black arrow) in contrast to the assay with the FA2 fragment carrying two *sps* sites in which several retarded bands were detected (Figure 2A). These differences probably reflect SpdA2-His binding to a single *sps* or to both *sps* sites.

The *sps* repeats of plasmid pSVH1 differ from the pIJ101 *sps* repeats in that they contain a T insertion. To study whether SpdA2 is also able to interact with the pSVH1-*sps*, we performed an EMSA using SpdA2-His and DNA fragments FSpdA, a PCR product of the coding region of *spdA* of pSVH1 which lacks the palindromic sequence (Figure 2E), and FSpdA_up_, a longer product that includes the upstream region in which the *sps* repeats are present (Figure 2D). Only the FSpdA_up_ fragment was shifted in the presence of SpdA2-His (Figure 2D, black arrows). Since the retarded fragments FSpdA_up_ and FA2 did not show any significant similarity beside the palindromic sequences, it is further evidence that the palindrome alone is critical for SpdA2 binding.

### *spdA2* is not essential for plasmid replication

There are contradicting reports concerning the function of *spdA* genes. Inactivation of *spdA* in plasmid pJV1 had no striking phenotype (Servín-González et al., 1995), while the SpdA2 encoding region of pIJ101 has previously been reported to be involved in segregational stability (Kieser et al., 2000). In contrast, *spdA* of pSN22 was shown to have a role in intramycelial plasmid spreading and pock formation (Kataoka et al., 1991). All these data were achieved by quite non-specific insertion/deletion analyses of *Streptomyces* plasmids (Kataoka et al., 1991; Servín-González et al., 1995) and do not allow to draw a final conclusion on the SpdA/SpdA2 function.

To elucidate the role of SpdA2, we deleted *spdA2* from the conjugative pIJ101 derivative pIJ303 (pIJ303Δ spdA2), avoiding any polar effects on the downstream single-stranded origin *sso*, involved in rolling-circle replication, and allowing easy complementation with different constructs (Figure 3). The *spdA2* defect was complemented by the insertion of a NdeI/HindIII fragment encoding SpdA2-His (pIJ303spdA2c). To characterize the molecular function of *sps*, we also complemented plasmid pIJ303Δ spdA2 by the insertion of fragment FA2-sps, yielding pIJ303-sps (Figure 3). In this fragment the palindromic *sps* was removed from the *spdA2* sequence by specific base substitutions that did not affect the SpdA2 amino acid sequence.

All plasmids could be introduced into *S. lividans* by PEG mediated protoplast transformation with similar efficiency, demonstrating that neither *sps* nor *spdA2* had an essential role in plasmid replication.

### Deletion of *spdA2* or *sps* does not interfere with conjugative plasmid transfer and pock formation

To study the presumed role of *spdA* in conjugative transfer, *S. lividans* TK54 carrying pIJ303 and its mutated derivatives were used as donors in mating experiments with *S. lividans* TK64::pSET152, containing pSET152 integrated into the PhiC-31 attachment site as a recipient. Frequency of conjugative transfer was calculated by mixing approximately 10^7^ spores of plasmid carrying donor and plasmid free recipients on R5. Under these mating conditions the effect of intramycelial plasmid spreading should be negligible. In these matings, all plasmids were transferred with statistically similar frequencies of 95–100% (Table 3).

**Table 3:** **Transfer frequencies of the pIJ303 derivatives**.

**Plasmid**	Transfer frequency (transconjugant/recipient) (%)^*^				
	**1**	**2**	**3**	**4**	**Mean ± *SD***
pIJ303	93.83	103.18	101.03	95.90	95.83 ± 1.57
pIJ303ΔspdA2	97.49	96.48	95.77	92.92	100.20 ± 4.04
pIJ303-sps	95.43	96.95	92.74	98.16	96.35 ± 3.44
pIJ303spdA2c	96.55	104.19	95.87	106.21	98.30 ± 5.69

* *Values of four biological replicates, no statistically significant difference; SD, standard deviation*.

**Figure 4:**
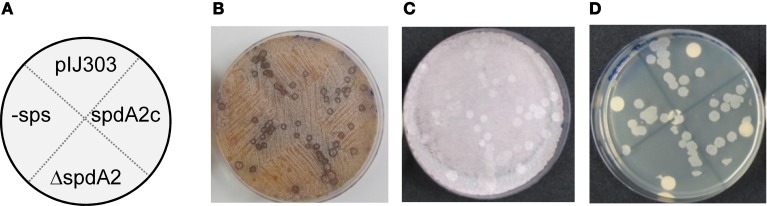
**Genetic crosses to visualize transfer and intramycelial plasmid spreading of pIJ303 and its derivatives**. Dilutions of *S. lividans* TK54 containing derivatives of plasmid pIJ303 (thio^R^) were streaked on a lawn (~10^6^ spores) of plasmid free TK64::pSET152 (apra^R^) on R5 plates, as schematically illustrated **(A)**. Pock structures associated with the conjugative plasmid transfer were visible after 2 days of incubation at 30°C **(B)**. After 7 days of incubation the fully sporulated plate **(C)** was replica plated on LB containing apramycin and thiostrepton to select for transconjugants **(D)**. Pock diameters and sizes of the transconjugant areas indicating efficiency of conjugative transfer and intramycelial plasmid spreading were approximately the same for all derivatives of pIJ303.

To assess the effect of *spdA2* deletion on pock formation indicating intramycelial plasmid spreading, ~10^2^ donor spores were streaked onto a lawn (~10^6^) of recipient spores. After 2 and 7 days incubation, plates were analyzed for pock formation (Figure 4). Finally, the sporulated plates were replica-plated on antibiotic-containing agar to select for transconjugants. In these experiments all pIJ303 derivatives produced pocks of identical diameter (Figure 4B). Moreover, transconjugant patches growing after replica-plating on selective agar had the same size for all constructs (Figure 4D). These data show that *spdA2* is not crucial for intramycelial plasmid spreading. If SpdA2/*sps* would have a role in conjugative transfer and subsequent spreading, pock sizes and morphologies should have been affected by the respective deletions.

### *spdA2* and *sps* affect segregational stability of plasmid pIJ303

To analyse segregational stability, frequency of plasmid loss was determined by plating spores after one, two and three cycles of non-selective sporulation at 30°C. Subsequently the colonies were transferred to thiostrepton containing media, to calculate loss of the plasmid encoded thiostrepton resistance (Table 4). While plasmids pIJ303 (100%) and pIJ303spdA2c (100%) were stably maintained even after three rounds of non-selective sporulation, 16 and 21%, of the colonies had lost pIJ303ΔspdA2 and pIJ303-sps, respectively. The similar ratio of plasmid loss demonstrates that segregational stability of pIJ101 is equally affected, irrelevant whether the DNA binding protein SpdA2 or its target sequence *sps* was inactivated.

**Table 4:** **Influence of *spdA2/sps* on plasmid stability (percentage of plasmid content)**.

**Plasmid**	**1. Sporulation cycle**	**2. Sporulation cycle**	**3. Sporulation cycle**
pIJ303	99.75 ± 0.50	100 ± 0	100 ± 0
pIJ303ΔspdA2	100 ± 0	94.50 ± 0.57^*^	83.50 ± 5.92^*^
pIJ303-sps	99.25 ± 0.96	95 ± 4^*^	79 ± 6.25^*^
pIJ303-spdA2c	99.50 ± 0.58	100 ± 0	99.75 ± 0.50

*Mean of four repetitions ±: standard deviation*,

* *significant when p < 0.001*.

Localization of SpdA2/*sps* between *rep* and the single-stranded origin *sso* suggested a functional connection, e.g., a role in the conversion of single-stranded plasmid intermediates resulting from rolling-circle replication into double-stranded DNA. Therefore, the amounts of single-stranded plasmid accumulating in *S. lividans* strains carrying plasmids pIJ303, pIJ303Δ spdA2, pIJ303-sps, and pIJ303spdA2c was studied and compared to that of plasmid pIJ702 which lacks a functional *sso*. Protoplasts containing the respective plasmids were lysed in the gel pockets of an agarose gel and the released plasmid DNA was separated. After blotting onto a nylon membrane without previous denaturation, the single-stranded plasmid was visualized with a digoxigenin labeled probe (see Materials and Methods). Whereas single-stranded plasmid intermediates were readily detected for plasmid pIJ702, neither pIJ303, nor pIJ303-sps, pIJ303Δ spdA2, or pIJ303spdA2c accumulated significant amounts of single-stranded DNA (Figure 4). Therefore, the effect of SpdA2/*sps* on plasmid stability is not caused by stimulating the conversion of single-stranded plasmid molecules into double-stranded DNA.

**Figure 5:**
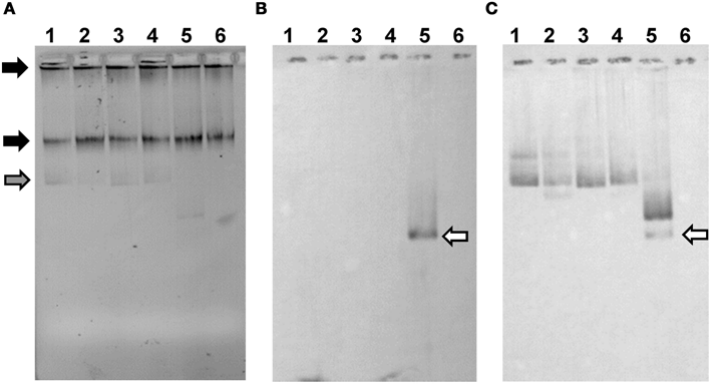
**pIJ303 Δ spdA2 and pIJ303-sps do not accumulate single-stranded plasmid DNA**. Approximately 10^6^ protoplasts of *S. lividans* TK54 with respective plasmids were lysed in a 1% TA agarose gel containing 0.25% SDS **(A)**. Black arrows indicate chromosomal DNA, while the gray arrow marks ccc-DNA. The gel was either blotted in native state **(B)** to detect accumulation of single-stranded plasmid DNA, or denatured before blotting in order to detect also double-stranded plasmid DNA **(C)**. As a probe a 600 bp PCR-fragment corresponding to the *dso* of pIJ101 was used. Plasmid pIJ702, which lacks the *sso*, was used as a positive control for the accumulation of single-stranded plasmid DNA (white arrow). 1, pIJ303; 2, pIJ303-sps; 3, pIJ303Δ spdA2; 4, pIJ303spdA2c; 5, pIJ702; 6, plasmid free TK54.

The in-gel lysis procedure suggested similar copy numbers for pIJ303, pIJ303Δ spdA2, pIJ303-sps, and pIJ303spdA2c (Figure 5A). To compare the copy numbers of the pIJ303-derivatives more accurately, we performed qPCR on total DNA extracted from equal amounts of mycelium. To detect plasmid DNA we amplified a 146 bp fragment of the thiostrepton resistance gene *tsr*. As a chromosomal reference a 146 bp fragment of the *S. lividans xre* gene was amplified. PCR efficiencies and Ct values were used to calculate the relative quantity of the different plasmid derivatives according to Pfaffl (2001). The mean values (±standard deviation) of five biological replicates were 1.00 (pIJ303), 0.82 ±0.27 (pIJ303Δ spdA2), 1.02 ±0.43 (pIJ303-sps), and 0.97 ±0.49 (pIJ303-spdA2c). Because these values did not show any statistically significant differences, the effect of *spdA2/sps* on plasmid stability is probably not caused by alterations in the plasmid copy number.

## Discussion

Since, with few exceptions, *Streptomyces* plasmids usually show only weak sequence similarities (Vogelmann et al., 2011a), identification of the 12-bp palindromic *sps* motif on many *Streptomyces* plasmids was a surprise. *sps* is always associated with *spdA* which encodes a protein of 117–163 amino acids. SpdA proteins show higher sequence conservation only within the N-terminal 50 aa, corresponding to the putative DNA binding domain. Plasmids that lack a *spdA* gene, like pSG5 (Maas et al., 1998) also do not contain *sps* repeats. The conservation of *sps* not only strengthens the relevance of its biological role but it might also indicate that SpdA/SpdA2 and *sps* may interact with chromosomal encoded functions that are well-conserved in different *Streptomyces* species. Despite being a quite short sequence, *sps* is only rarely found on chromosomes. The CGTCACGTGACG sequence occurs a single time in the genome of *S. coelicolor* (upstream of SCO4580).

Although *spdA* homologous genes have been characterized before, no conclusive function could be assigned. While disruption of *spdA* in the *S. nigrifaciens* plasmid pSN22 led to a reduced pock size, inactivation of the pJV1 homolog did not cause a striking phenotype (Kataoka et al., 1991; Servín-González et al., 1995). On plasmid pIJ101 the SpdA homolog SpdA2 might contribute to plasmid stability, since a deletion of the pIJ101 region containing *spdA2* has been reported to affect segregational stability, although the structurally and segregationally stable pIJ680 lacks this region (Kieser et al., 2000). Localization of *spdA* of all rolling-circle replicating plasmids between the replication initiator gene *rep* and the single-stranded origin *sso* suggests a functional relationship. The *sso* origin of pIJ101 has been demonstrated to affect plasmid copy number and plasmid maintenance (Deng et al., 1988). The minus origin *sso* is required for the efficient conversion of the single-stranded replication intermediate into a double-stranded plasmid molecule by recruiting host factors (Kramer et al., 1997). For plasmid pSN22 a RNA polymerase independent priming mechanism for the conversion of the single-stranded replication intermediate into double-stranded plasmid DNA has been reported (Suzuki et al., 2004). However, attempts to detect an influence of *spdA2/sps* on the efficiency of single-stranded DNA conversion failed in this study. Only pIJ702 lacking a functional *sso* accumulated significant amounts of ss-DNA, whereas deletion of *sps* or *spdA2* of plasmid pIJ303 did not interfere with the conversion of ss-DNA into ds-DNA. Furthermore, presence of a *spdA* gene next to *traB* in plasmid pSG2 (Wohlleben and Pühler, 1987) which does not encode a replication initiator protein for RCR and does most probably replicate via a theta mechanism argues against a role of *spdA* in rolling-circle replication.

Segregational instability of a multi-copy plasmid could be also caused by a reduction in copy number. But determination of the relative plasmid copy numbers of the different pIJ303 derivatives did not reveal a statistically significant difference. Therefore, *spdA2/sps* do not seem to be involved in the copy number control of pIJ101.

As it was suggested by the association of *sps* with the presence of the putative DNA binding protein SpdA, SpdA2 of pIJ101 recognized the palindromic *sps*. Appearance of several retarded bands in EMSAs (Figure 2) probably reflects binding of SpdA2 to one or both palindromic sequences. Recognition of a single palindromic sequence is in agreement with the occurrence of a single palindrome in *spdA* of pTSC1 and the presence of even three palindromic sequences in plasmid pSG2, pSNA1, pEN2701, or pSN22 (Figure 1). However, the different retarded bands could also indicate that SpdA/SpdA2 might bind as a dimer or oligomer, as it is often seen with DNA binding proteins (Zhu and Huq, 2011).

Interestingly, SpdA2 of pIJ101 was also able to bind to *sps* of plasmid pSVH1, although *sps* of pSVH1 has a T insertion in the center of the palindrome (Figure 1B). This clearly indicates that despite the high variability of the SpdA proteins of different plasmids they all interact with the same target sequence *sps*.

### What might be the role of SpdA in plasmid biology?

Occurrence of SpdA homologs only on *Streptomyces* plasmids implicates SpdA/*sps* with a specific function caused by the distinct *Streptomyces* life style, which involves growth by apical tip extension, hyphal branching and morphological differentiation (Flärdh, 2003). This might include distinct positioning of the plasmids within the mycelium and transport of plasmid copies over large distances during hyphal branching. The proposed interaction with chromosomally encoded proteins involved in replication, segregation or cytoskeletal structures would explain the high level of conservation of *sps* sequences in different plasmids.

Although we demonstrated the specific interaction of SpdA2 with *sps*, inactivation of *spdA2* or *sps* in pIJ303 had only a mild phenotype. Transfer frequency as well as the size of the formed pocks was not affected. Only after two cycles of sporulation, segregational stability was significantly impaired. This is in agreement with the previous report of Kieser et al. (2000) and the genetic localization between *rep* and *sso*. The mild phenotype of SpdA2/*sps* deletion suggests that SpdA2/*sps* is active only under undetermined environmental conditions not replicated in laboratory assays or compensated by redundant stability systems. Also deletion of *par* of the linear plasmid SLP2 affected stability only slightly and caused 14% plasmid loss after two cycles of sporulation (Hsu and Chen, 2010).

But why have *spdA* genes of other plasmids been characterized as spreading function affecting pock formation (Kataoka et al., 1991) or without any phenotype (Servín-González et al., 1995)? Due to the mycelial growth of *Streptomyces*, plasmid stability and intramycelial plasmid spreading cannot be seen separately. On the one hand, plasmid instability will affect plasmid spreading by decreasing the number of plasmid molecules to be transferred. But plasmid spreading can be also considered as a stability function. Intramycelial plasmid spreading provides a route allowing mycelial fragments that have lost the plasmid due to segregational instability to regain the plasmid from the neighboring compartment. Therefore, *spd* genes involved in plasmid spreading might also have a role in plasmid stability. Enhanced instability of a *spdB2* mutant and a stabilizing effect of *spdB2* has been reported for of the linear plasmid SLP2 (Hsu and Chen, 2010).

Even though more work is necessary to fully understand the molecular function of SpdA proteins, we have provided clear evidence that both SpdA and its binding site *sps* might play an important role in the biology of *Streptomyces* plasmids.

## Author contributions

Conceived and designed the experiments: Lina Thoma, Edgardo Sepulveda, Günther Muth. Performed the experiments: Lina Thoma, Annette Latus, Edgardo Sepulveda. Analyzed the data: Edgardo Sepulveda, Lina Thoma, Günther Muth. Wrote the paper: Edgardo Sepulveda, Lina Thoma, Günther Muth.

### Conflict of interest statement

The authors declare that the research was conducted in the absence of any commercial or financial relationships that could be construed as a potential conflict of interest.
